# Persistent Fetal SVT in a COVID-19 Positive Pregnancy

**DOI:** 10.1155/2022/9933520

**Published:** 2022-01-07

**Authors:** Gloria Wang, Eric Stapley, Sara Peterson, Jessica Parrott, Cecily Clark-Ganheart

**Affiliations:** ^1^Overland Park Regional Medical Center. HCA Graduate Medical Education-ObGyn, 12200 W. 106th St, Suite 325, Overland Park, KS 66215, USA; ^2^Kansas City University of Medicine and Biosciences, 1750 Independence Ave, Kansas City, MO 64106, USA; ^3^Midwest Perinatal Associates, 12200 W. 106th Street #110, Overland Park, KS 66125, USA; ^4^Perinatal Specialists of Kansas City, 16240 Foster St, Overland Park, KS 66085, USA

## Abstract

**Background:**

Rapid introduction and spread of SARS-CoV-2 have posed unique challenges in understanding the disease, role in vertical transmission, and in developing management. We present a case of a patient with COVID-19 infection and fetus with new-onset fetal SVT.

**Case:**

A 26-year-old gravida 4 para 2012 with third trimester COVID-19 infection was diagnosed with new onset fetal SVT. Successful cardioversion was achieved with flecainide. The patient was followed outpatient until induction of labor at 39 and 3/7 weeks of gestational age resulting in an uncomplicated vaginal delivery. Postpartum course was uncomplicated.

**Conclusion:**

Fetal SVT is a potential complication of maternal COVID-19 infection. The use of transplacental therapy with flecainide is an appropriate alternative to digoxin in these cases.

## 1. Introduction

Coronavirus disease 2019 (COVID-19), first identified in December 2019 in Wuhan, China, is caused by the severe acute respiratory syndrome coronavirus 2 (SARS-CoV-2). To date, COVID-19 cases in the world surpassed 200 million with over 5 million deaths [1]. Over 18,000 pregnant women in the US have been infected, with pregnancy serving as a risk factor for severe illness [2]. Initial studies were limited regarding the full clinical significance on perinatal and fetal populations [3, 4]. Later studies showed that, compared to uninfected pregnant women, women with COVID-19 had increased rates of preeclampsia, preterm birth, venous thromboembolism, mortality, need for intubation, and ICU admissions [5].

Vertical transmission of COVID-19 appears to be rare, but possible. In pregnant women with positive COVID-19 test or clinical diagnosis of COVID-19, the majority of cases were negative for COVID-19 in samples including breastmilk, amniotic fluid, umbilical cord blood, and neonatal throat swabs. In the rare cases with positive results, variations in collection times and other factors could not rule out postnatal infection [3, 4, 6]. One case report describes a positive test in breastmilk [4]. Another report describes a newborn with transient neurological compromise who had positive samples alongside positive tests in placental and amniotic fluid samples [7].

In this report, we present a case of a 26-year-old COVID-19-positive patient in a fetus with new onset supraventricular tachycardia (SVT) treated with flecainide. While intermittent SVT poses a low risk of hemodynamic complications, sustained SVT (over 50% of fetal monitoring time) can result in congestive heart failure leading to nonimmune fetal hydrops, premature delivery, and fetal demise [8, 9]. After extensive literature review to date, this is the first known case of fetal arrhythmia associated with COVID-19 infection.

## 2. Case Presentation

**Figure 1:**
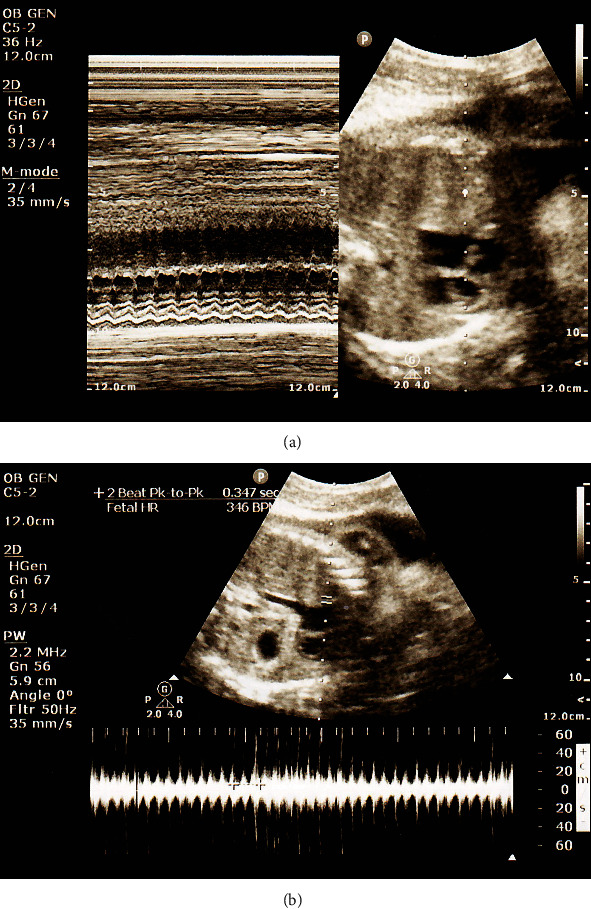
Bedside ultrasound on day of admission, demonstrating fetal tachycardia on (a) M-mode and (b) spectral Doppler mode.

A 26-year-old G4P2012 female was transferred from an outside hospital at 31 and 5/7 weeks of gestation for fetal tachycardia. Her pregnancy was complicated by a positive COVID-19 test one day prior to transfer, tobacco use, and GBS positive status. Three days prior to transfer, she reported anosmia and ageusia. She was otherwise healthy. On admission, vitals were stable and baseline CMP and CBC were appropriate for pregnancy. Fetal nonstress test (NST) was significant for fetal intermittent SVT to 170-200 s bpm over 50% of the time. Bedside ultrasound demonstrated a 1 : 1 ratio with fetal heart rate of 340 bpm. Growth was appropriate for gestational age with normal Doppler studies and AFI (Figure 1). Pediatric cardiology was consulted, and the patient was started on oral digoxin with initial improvement in fetal heart rate and frequency. Fetal echocardiogram did not review structural abnormalities. However, on hospital day 3, SVT reoccurred with persistent rate of 230 bpm. Digoxin was converted to flecainide on hospital day 4 with improvement in frequency and severity of fetal SVT with intermittent rates of 170-180 s. She was discharged home on flecainide and 162 mg of aspirin. The patient followed up on an outpatient basis with biweekly biophysical profile (BPP) scans and monthly growth ultrasounds. Outpatient monitoring demonstrated resolution of SVT without evidence of hydrops. Flecainide was discontinued the week prior to delivery. The patient underwent scheduled induction of labor at 39 and 3/7 weeks of gestation via oxytocin. She had an uncomplicated spontaneous vaginal delivery of a 3565 g infant with Apgar scores of 8/9/9.

## 3. Discussion

The patient in our case report is a unique instance of fetal SVT in the presence of maternal COVID-19 infection. It is possible that vertical transmission has the ability to affect cardiac conduction in the fetus. Much of the initial information in understanding COVID-19 relied on comparisons with similar betacoronaviruses, including SARS and MERS, which did not show evidence of vertical transmission in pregnancy and demonstrated variable effects on pregnancy complications [3, 10]. Recent evidence for SARS-CoV-19 has shown that the virus infects cells via the angiotensin-converting enzyme 2 (ACE) receptor present on respiratory epithelium. ACE2 receptors are expressed in the placenta with some placental samples demonstrating fibrin deposition involving villi [11]. Despite this, cases of possible vertical transmission are few [6, 12].

This case may be a new and rare association between betacoronaviruses and fetal arrhythmias in general. Fetal SVT is one of the most common fetal arrhythmias resulting in fetal heart rates over 200 bpm [13]. SVT is often caused by reentrant tachycardia, thought to be initiated through a spontaneous premature atrial complex (PAC) as well as a response to maternal or fetal infection [9]. Although fever can trigger episodes of SVT, our patient did not endorse fevers and was afebrile during her hospital stay. To our knowledge, previous cases of fetal arrhythmias in pregnancies complicated by SARS or MERS have not been reported. However, other maternal infections, such as influenza and urosepsis, have reported cardiac arrhythmias [14, 15]. In adults with influenza, arrhythmias are hypothesized to be secondary to systemic, arterial, and myocardial inflammation, as well as increased metabolic demand, hypoxia, and adrenergic responses. In adult COVID-19 patients, additional potential causes include effects on myocardial and pulmonary ACE2 pathways and cytokine storms [16]. Therefore, the true effect of COVID-19 on fetal arrhythmias may be a combination of these factors.

Transplacental treatment of fetal SVT has some variability. When fetal heart rate is over 180 bpm, treatment is warranted to avoid the complications of heart failure, hydrops, and subsequent morbidity and mortality [17]. While flecainide and sotalol have shown to be more effective than digoxin when hydrops is present, the data is less consistent in the absence of hydrops. Digoxin, flecainide, and sotalol have been supportive as effective treatments for SVT without hydrops, and amiodarone has been used for treatment-resistant tachycardia [17, 18]. Miyoshi et al. developed a one-armed protocol by treating first with digoxin, then sotalol, and third flecainide with resolution of SVT in 89.8% of nonhydrops cases [8]. Ekiz et al. have shown similar effectiveness with flecainide [19]. Management of patients on these medications through monitoring maternal drug levels is variable as well [20]. In our circumstance, prompt resolution of fetal SVT and maternal medication tolerance allowed us to provide outpatient management.

Management of COVID-19 and SVT during pregnancy depends on the stability of the mother and fetus and the complications present. An interdisciplinary coordination and assessment of risks and benefits is crucial. Further studies are necessary to explore the extent fetal complications of COVID-19, including the potential association of fetal arrhythmias.

## Data Availability

The case report relies on protected health information owned by HCA; thus, the individual chart will not be accessible to readers.
